# Model-based QTL detection is sensitive to slight modifications in model formulation

**DOI:** 10.1371/journal.pone.0222764

**Published:** 2019-10-03

**Authors:** Caterina Barrasso, Mohamed-Mahmoud Memah, Michel Génard, Bénédicte Quilot-Turion

**Affiliations:** 1 GAFL, INRA, 84143, Montfavet, France; 2 PSH, INRA, 84914, Avignon, France; University of North Carolina at Greensboro, UNITED STATES

## Abstract

Classical crop models have been developed to predict crop yield and quality, and they are based on physiological and environmental inputs. After molecular discoveries, models should integrate genetic variation to allow predictions that are more genotype-dependent. An interesting approach, Quantitative Trait Locus (QTL)-based ecophysiological modeling, has shown promising results for the design of ideotypes that are adapted to biotic and abiotic stresses, but there are still limitations to attaining a fully integrated model. The aim of this case study is to clarify the impact of choosing different model equations (closely related and with different numbers of parameters) and optimization methods on the detection of QTLs controlling the parameters of crop growth. Different growth equations were parameterized based on a genetic population by following different approaches. The correlations between parameters were analyzed, and two different strategies were adopted to address the correlation issue. QTL analysis was performed on the optimized values of the parameters of the growth equations and on the observed dry mass (DM) data to validate the QTLs detected. Overall, models and strategies resulted in different QTLs being detected. Similar LOD profiles but with peaks of different heights were observed, some of which were significant, resulting in different numbers of QTLs. In some cases, peaks had slightly different positions or were absent. Even closely related growth models led to the detection of different QTLs. The goodness of fit and complexity of the growth models were found to be insufficient to select the best model. Calculating parameters independently of observed data may not be a good strategy, whereas setting parameters independent of the genotype is recommended. Given the large-scale global optimization problem and the strong correlations between parameters, the two algorithms tested showed poor performance. Currently, the lack of effective algorithms is the main obstacle to answering the question posed. The authors therefore suggest testing different model formulations and comparing the QTLs detected before choosing the best formulation to use in an ecophysiological modeling approach based on QTLs.

## Introduction

An ambitious goal for the future in agriculture is to design sustainable production systems that are environmentally friendly and produce quality food. To help achieve this objective, classical crop models have been designed to predict crop yield and quality in fluctuating environments [1]. Most of the agricultural phenotypes in nature are quantitative and thus are determined by the effect of quantitative trait loci (QTLs) in combination with environmental factors. The challenge for the agricultural research field is to find the best combination of genetic resources and cultural practices for target environments. Currently, classical crop models are still calibrated and developed for a few varieties of a specific species, restricting the application of classical models to the study of genotype-by-environment interactions on crop phenotypes. Consequently, future crop models are progressively required to additionally describe genetic variations.

To allow the description of genetic variations, future crop models will require genetic parameters that are specific for each genotype and are constant under different environmental conditions [2–3]. Each parameter should then be related to a set of interconnected processes controlled by a pool of genes. In the absence of information on specific genes or loci controlling the processes, QTL analysis can be performed on the physiological parameters of classical crop models. The latter can be considered as quantitative physiological traits, and their inheritance can be elucidated [4] [5]. The set of parameters is then amenable to QTL analysis with i) the identification of QTLs controlling values of the parameters and ii) the back injection of the QTL-based parameter values into the process-based model [5] in order to remove the noise of the original estimates. For each genotype of a mapping population, the crop phenotypes can thus be predicted based on the allelic composition of the molecular markers flanking the detected QTLs [5]. The resulting approach, QTL-based ecophysiological modeling, was first formulated and practiced as reported by Yin et al. [6] in barley.

The potential of the QTL-based ecophysiological modeling approach is the capacity to predict the behavior of many genotypes under different environments, to design ideotypes adapted to biotic and abiotic stresses, to test hypotheses on likely mechanisms and to guide research and accelerate scientific understanding [3, 7–9]. Two main approaches were found in the literature to ensure the applicability of QTL-based ecophysiological modeling. The first is called functional mapping, defined as the top-down approach by Wei et al. [10]; it integrates mathematical relationships of different traits into QTL mapping theory. Various authors have applied functional mapping [11–14], using different mathematical relationships. As an example, Hou et al. [15] used a maximum-likelihood approach based on a logistic-mixture model and implemented with the EM algorithm. Chang-Xing et al. [16] applied functional mapping with nonlinear mixed-effect models, while Xing et al. [17] used Bayesian B-spline mapping. A second approach, defined as the bottom-up approach by Wei et al. [10], is preferred by several authors [18–20]. The method consists of fitting the models first, followed by mapping QTLs based on the estimated parameters. It is usually employed when the process-based model used is complex and nonlinear.

One of the main advantages of functional mapping is the stability of the method by modeling the patterns of trait development and autocorrelations among different time points. This may improve the statistical power to detect significant QTLs [11]. Biological models usually contain a large number of parameters among which correlations exists [21–22]. Even a good fit cannot guarantee unique parameter estimation, therefore finding the true parameter value remains a challenge since it is hidden in the correlated relations [23–24]. Finally, the method can also outperform the bottom-up approach in terms of curve estimation precision [10].

Despite the promising properties, some authors have identified disadvantages in the functional mapping approach. First, the combination of a complex process-based model together with the QTL mapping theory may not be feasible. Additionally, the expected values for different QTL genotypes at all time points and for all elements in the covariance matrix need to be estimated, resulting in substantial computational difficulties. Similarly, the bottom-up method can demonstrate high power for QTL detection if the model is correct, and it allows for efficient analysis of unbalanced phenotypic data. Kwak et al. [25] showed that it has reasonable power to detect QTLs in many situations.

In addition to the choice of the approach to detect QTLs of parameters, an important knowledge gap for the development of a QTL-based ecophysiological modeling approach is to quantify the influence of the crop model choice on the detection of QTLs. In other words, it is important to know if the QTLs detected in relation to a modeled process are extremely dependent on the choice of the model equation used to represent the process. To provide answers to this question, a case study was conducted to investigate the effect of using different, but very closely related, simple models (parameterized on a same dataset) on the detection of QTL controlling parameters. For the sake of simplicity and considering that the conclusions can be extended to complex models, this study focused on growth equations. They constitute a good study case because a large number of similar equations have been published and the parameters of these equations are, for the most part, highly correlated. With the idea of using the conclusions and applying recommendations of this study for the development of a QTL-based ecophysiological modeling approach with a very complex model [26], and given the more direct application of the bottom-up approach compared to functional mapping (considering also our own expertise), we elected to follow the former in our case study. By using this approach, autocorrelations between values at different time points are not modeled. We studied correlations between the parameters of the equations and compared different models with ‘low’ and ‘high’ (when most of the correlations are observed) numbers of fitted parameters to investigate the influence of over-parameterization on QTL detection. We also explored two strategies targeting the correlation issue: i) parameterization of few parameters as genotype-independent ii) replacement of parameters adjusted with observed values. Finally, we used two optimization algorithms for the estimation of growth model parameters.

**Fig 1:**
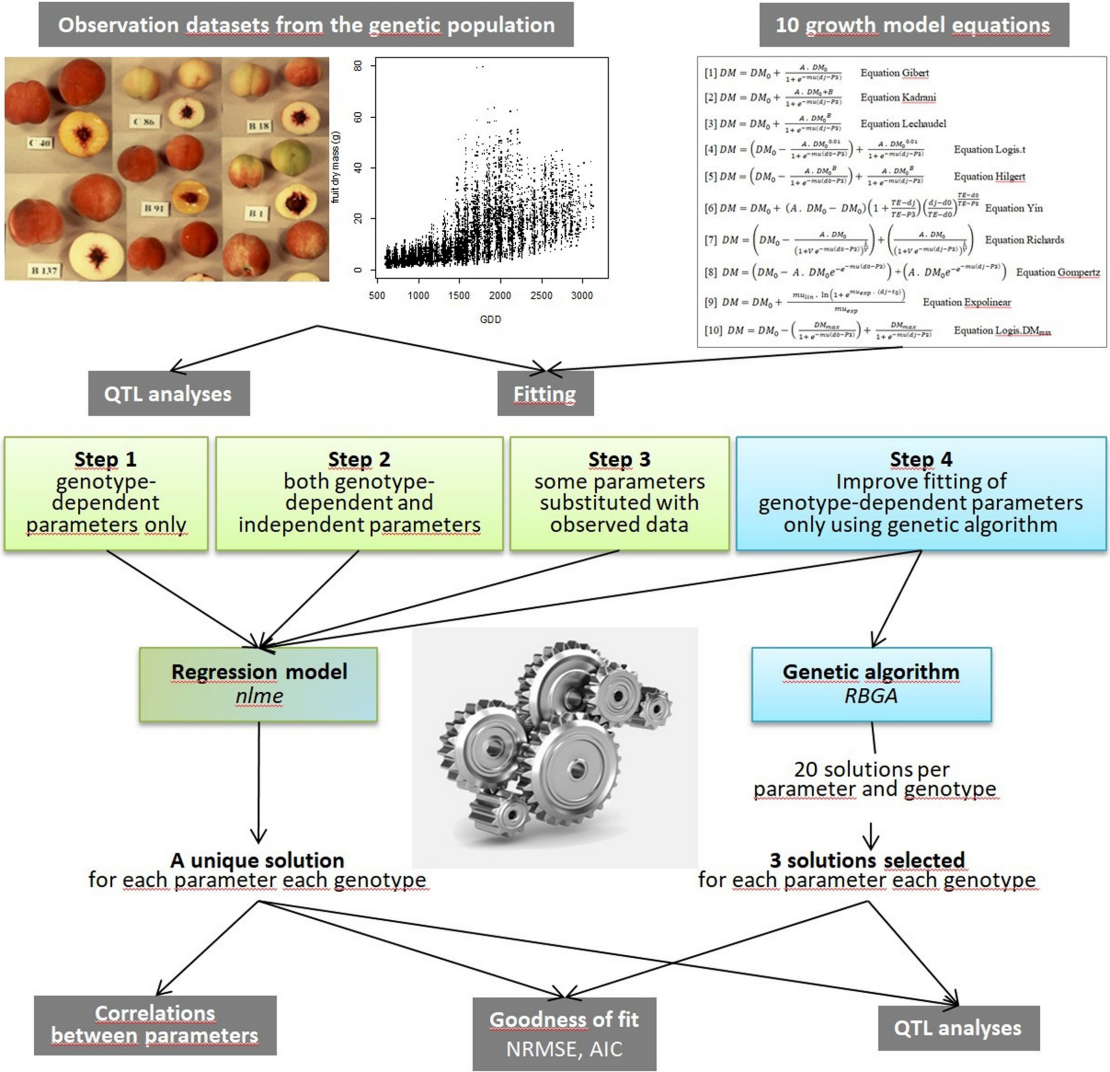
Schematic representation of the approach developed during this study. The experiment was conducted with an observation data set of fruit DM available for the genetic population of peach. Ten growth models were parameterized on the population by use of nlme (Steps 1–3) and RBGA (Step 4) algorithms. Solutions with nlme were used to study correlations between parameters. The goodness of fit of each solution was calculated. Solutions obtained with the two algorithms and the observed data (final fruit DM and mean growth rate) were amenable to QTL analyses.

The case study was conducted with a peach population described by Quilot et al. [27]. Data for dry mass (DM) were available and different biological growth models were parameterized using classical and evolutionary algorithms. QTL analysis was performed on the optimized values of the parameters and on the observed DM data to validate the QTLs detected. The description of the methodology adopted during the study is shown in Fig 1.

## Materials and methods

### Genetic material and phenotypic data

The genetic population of peach used in the study is described by Quilot et al. [27]. A *Prunus davidiana* clone (P1908) and *Prunus persica* cv. ‘Summergrand’ were crossed. The first back crossed population (F1) obtained was then back crossed to *Prunus persica* cv. ‘Summergrand’ to obtain the first back crossed population. A pollen mixture from F1 hybrids was then used to fertilize another commercial peach variety, Zephir, to obtain the second pseudo-backcrossed population. In our study, one hundred and sixty-one genotypes from the second pseudo-backcrossed population and the parental genotypes *Prunus persica* cv. ‘Summergrand’, Zephir and *Prunus davidiana* clone P1908 were used for estimation of parameters of the growth models analyzed. As described by Quilot et al. [27], the population was grown under normal conditions of irrigation, fertilization, and pest control. Environmental sources of variation between genotypes and between fruits within a genotype were minimized by carrying out heavy fruit thinning. For one hundred and sixty-one genotypes individuals of the progeny and the three ancestors (‘Summergrand’, Zephir, and *Prunus davidiana* clone P1908), a fruit diameter check was conducted once a week. Then, to compute the DM, several allometric relationships between DM and diameter were used for each genotype [28]. For each genotype, the DM was calculated over time, and it was expressed in a growing degree days after flowering (GDD). Thus, in our study and for each genotype, DM was available for one to six fruits and for seven to twenty-two points in time. From this dataset obtained during the study, the average fruit DM at maturity (final fruit DM) and the average ratio between fruit DM and total GDD at maturity (mean growth rate) were calculated per genotype on the observed data to validate the QTLs detected with the parameters of the growth models.

### Growth models

To reach the goal of our study, very closely related growth models with intentionally ‘high’ and ‘low’ numbers of parameters were compared with different growth models. Ten sigmoidal growth models were considered to study the effect of the equation choice on the detection of QTLs that control the parameters of the equations. Most of the equations used during the study intentionally involve a ‘high’ number of parameters in order to answer the research questions of this case study. Most of those equations are derived from the logistic function [29]:

$$\text{DM}=\frac{DM_{max}}{1+e^{-mu(dj-P3)}}$$ (1)

where *DM* is dry mass, *DM*_*max*_ is the final fruit dry mass, and *dj* is the time expressed in GDD. The remaining parameters are described in (Table A in S1 File).

The ten growth models used during the study consider the impact of initial fruit dry mass *DM*_*0*_ (observed value) on *DM*_*max*_, and they encompassed differing numbers of parameters (from two to four). Some of our growth equations are the most commonly used models [10].

$$DM=DM_{0}+\frac{A\cdot DM_{0}}{1+e^{-mu(dj-P3)}}$$ (2)

$$DM=DM_{0}+\frac{A\cdot DM_{0}+B}{1+e^{-mu(dj-P3)}}$$ (3)

$$DM=DM_{0}+\frac{A\cdot DM_{0}{}^{B}}{1+e^{-mu(dj-P3)}}$$ (4)

$$DM=\left(DM_{0}-\frac{A\cdot DM_{0}{}^{0.01}}{1+e^{-mu(d0-P3)}}\right)+\frac{A\cdot DM_{0}{}^{0.01}}{1+e^{-mu(dj-P3)}}$$ (5)

$$DM=\left(DM_{0}-\frac{A\cdot DM_{0}{}^{B}}{1+e^{-mu(d0-P3)}}\right)+\frac{A\cdot DM_{0}{}^{B}}{1+e^{-mu(dj-P3)}}$$ (6)

$$DM=DM_{0}-(A\;.\;DM_{0}-DM_{0})\left(1+\frac{TE-dj}{TE-P3}\right){\left(\frac{dj-d0}{TE-d0}\right)}^{\frac{TE-d0}{TE-P3}}$$ (7)

$$DM=\left(DM_{0}-\frac{A\cdot DM_{0}}{{\left(1+V\;e^{-mu(d0-P3)}\right)}^{\frac{1}{V}}}\right)+\left(\frac{A\cdot DM_{0}}{{\left(1+V\;e^{-mu(dj-P3)}\right)}^{\frac{1}{V}}}\right)$$ (8)

$$DM=\left(DM_{0}-A\;.\;DM_{0}e^{-e^{-mu(d0-P3)}}\right)+\left(A\;.\;DM_{0}e^{-e^{-mu(dj-P3)}}\right)$$ (9)

$$DM=DM_{0}+\frac{mu_{lin}.\ln\left(1+e^{mu_{exp}\cdot (dj-t_{0})}\right)}{mu_{exp}}$$ (10)

$$DM=DM_{0}-(\frac{DM_{max}}{1+e^{-mu(d0-p3)}})+\frac{DM_{max}}{1+e^{-mu(dj-p3)}}$$ (11)

Eq (2), is shown as eqGibert in this study and is the logistic function Verhulst modified by Gibert et al. [30] introducing a parameter, *A*, to express *DM*_*max*_ as being proportional to *DM*_*0*_. Eqs (3) and (4) are shown as eqKadrani and eqLechaudel [31] and are also modifications of the logistic function Verhulst, where another parameter, *B*, is introduced to express *DM*_*max*_ as linear and exponential forms of *DM*_*0*_ respectively. Eq (5) is shown as eqLogis.t and has parameter *B* fixed to a constant value (0.01) and *d*_*0*_ corresponds to time zero (full bloom) expressed in GDD. The equation was modified such that *DM = DM*_*0*_ at *dj = d*_*0*_. Eq (6) is shown as eqHilgert, and is a type of generalization of the modification of Eq (5) where parameter *B* is used to describe *DM*_*max*_ [32]. Eq (7) is shown as eqYin and was developed by Yin et al. [33–34]. It represents a symmetrical curve as a particular case of a flexible asymmetrical growth curve initially. Eqs (8) and (9) are shown as eqRichards and eqGompertz and are the asymmetrical growth curves proposed by Richards [35] and Gompertz [36], respectively, modified to start at *DM*_*0*_ (i.e., *dj = d*_*0*_). Eq (10) is shown as eqExpolin and is the expolinear growth model [37]. Eq (11) is shown as eqLogis.DM_max_ and is a modification of the logistic function Verhulst to start at *DM*_*0*_ and where the observed value of *DM*_*max*_ is explicitly included to have lower numbers of parameters to be estimated. Thus, the very closely related growth models, encompassing a different number of parameters, are Eqs (2–6) and (11). These equations have slight modification from the logistic function [29], and different number of parameters from ‘high’ to ‘low’ (respectively from four to two).

### Estimation of parameters

#### Fitting of growth models

Estimation of the genetic parameters for each growth model was executed by using the classical nonlinear mixed-effects models algorithm (nlme) in R from the package {nlme} and it is shown as **Step 1** in the study. The algorithm was used here to estimate all parameters for all genotypes of the population at the same time (with the same starting values) for each growth model. High correlations between parameters are often present in modeling studies. It is well known that when a smaller number of parameters has to be estimated, fewer correlations are observed. Therefore, other two strategies were adopted during the parameterizations with the nlme algorithm. These strategies attempted to reduce the number of parameters to be estimated and to study the effect of correlations between parameters on QTL detection.

The first one consisted in considering one or more parameters as genotype-independent (for Eqs from (2)–(10)), as reported in (Table B in S1 File) and indicated as **Step 2** in the study. The algorithm estimated those parameters as constant values within the genetic population, while specific values for each genotype were estimated for the other parameters. In this manner, it concentrated on the genotype-dependent parameters the entire genetic variability and reduced the number of estimated parameters per genotype.

Eq (11)) is the unique growth model explicitly involving the expression of *DM*_*max*_. Therefore, it was only used in the second strategy, which consisted of the replacement of parameter *P3* and the expression of *DM*_*max*_ with observed data values. For this strategy, indicated as **Step 3** in the study, the parameters were replaced by the average time of inflection point (*P3*) (estimated independently on observed data using the function *findiplist* (from the package {inflection} in R), and average final fruit DM calculated for each genotype on the observed data.

The choice of the nlme algorithm is motivated by its simplicity and its wide usage compared to other estimation methods, especially for maximizing the likelihood (see Comets et al. [38] for a comparison with SAEM: the stochastic approximation expectation maximization algorithm). However, being a linearization-based algorithm, nlme is known to have some issues with local optima even though it can demonstrate acceptable performance in some cases and allow at least initiating other fitting methods. Indeed, classical algorithms such as nlme could be ineffective for dealing with some difficult parameter estimation problems. Currently, more recent optimization algorithms are used to solve such problems, and they are employed to search parameter space by following a certain approach. Among different algorithms, nature-inspired optimization algorithms (e.g., genetic algorithms) are used with increasing frequency [39]. They are stochastic algorithms and, compared to gradient search techniques, are able to find global optimum values. A genetic algorithm (GA) was also used during the case study to explore other parameter spaces. Genetic algorithms have global search properties and are designed to deal with local optimum values, and they are considered to be powerful enough to deal with black-box optimization problems such as those seen in this study. Genetic algorithms can be used to improve the performance of other classical algorithms such as the expectation maximization algorithm [40]. Each GA has characteristics that influence its success, such as population size, crossover, selection, mutation operators and stopping criterion (e.g., maximum number of generations).

Model calibration was defined as a mono-objective optimization problem. The goal was to estimate the values of the genetic parameters that minimize the fitting errors in terms of fruit DM for each genotype. The R Based Genetic Algorithm (RBGA) from the package {GENALG} [41–42] was used, and it is shown as **Step 4** in the study. RBGA optimizes a set of floats using as input minimum and maximum values for the floats to optimize [43]. The performance (fitness) index used in the model calibration was the root mean square error (RMSE) summed for all fruits of each genotype. The optimal (fittest) is the chromosome (or vector of genetic parameters) for which the root-mean-square error is minimized. The settings for the RBGA algorithm used during the study are reported in (Table C in S1 File). To consider the stochasticity of the algorithm used and the uncertainties in estimated values, 20 simulations (repetitions) per growth model were run for a total of 200 simulations (10 growth models and 20 repetitions per growth model).

#### Multisolutions selected

To study the uncertainties in the solutions estimated with the RBGA algorithm, 3 of the 20 repetitions were selected for each growth model simulation and then used for the QTL analysis. The set of 3 parameters repetition selected for the study was:

Best: solution with the minimum RMSE (evaluation value) among 20 repetitions
Extreme 1 (extr.1) and extreme 2 (extr.2): the most different solutions among 20 repetitions

The most different solutions within each set of 20 repetitions were selected by using principal component analysis (PCA) with the function *dudi*.*pca* from the package {ade4} in R. For each growth model, each of the 161 genotypes and the three parental genotypes, a PCA was carried out on the 20 repetitions (20 solutions for parameter sets estimated by RBGA). The coordinates for each repetition of each genotype were collected on the two first principal components because most of the variation was explained by the first PCA plan. A distance matrix between each two-to-two repetition was compiled for each genotype and the two repetitions having a maximum distance were used as the most different solutions (extr.1 and extr.2) and used for the QTL-analysis. The extr.1 and extr.2 solutions always had the lowest and highest primary principal component values, respectively.

#### Goodness of fit

Because the goodness of fit of a model may have an influence on the detection of QTLs, the normalized root mean square error (NRMSE) and Akaike information criterion (AIC) were calculated inside the genetic population for each growth model with the nrmse function from the {hydroGOF} package and a user-built *function* in R, respectively.

The formula we used to calculate the AIC is the following:

AIC=n.log(SSE/n)−k.log(n) (12)

where SSE is the sum of squared errors, n is the number of observed data points, and k is the number of model parameters.

AIC is used to perform model comparisons and provides a trade-off between the goodness of fit and model complexity. Because it does not provide any information about the quality of a model, the NRMSE was calculated for each growth model to obtain the goodness of fit of the ten equations. The R function *aov* from the package {stats} was used to determine significant differences between NRMSE or AIC values obtained with the different strategies (3 solutions from nlme, (i.e., one by step) and 3 solutions from the RBGA algorithm). Differences were considered significant if *p* ≤ 0.05.

### QTL-Analysis

The genetic map (developed by Desnoues et al. [44]) monitoring the polymorphisms between *Prunus davidiana* and *Prunus persica* genomes was used. The peach is a diploid species, and the map used is composed of 340 informative genomic bins (markers) distributed across the 8 autosomal chromosomes of the peach. At any marker, there are two possible genotypes: QQ or Qq. For each phenotypic trait (estimated genetic parameter), the Shapiro-Wilk normality test was applied using the function ‘*shapiro*.*test’* from the package {stats} in R. Traits showing normal distributions were analyzed by interval mapping with the Haley–Knott regression method, as described by [45], using the ‘scanone’ function in the {rqtl} library in R. It consists of a regression of the phenotypes on the multipoint QTL genotype probabilities for having genotypes QQ or Qq at the putative QTL, given the marker data. The null hypothesis, H_0_, corresponds to no QTL and the alternative, H_1_, to the presence of a QTL.

Traits that did not show normal distributions were transformed by calculation of the natural logarithm and square root, then analyzed using a nonparametric model, as extension of the Krustal-Wallis test as described by [46].

The LOD score was calculated as follows:

$$\text{LOD}=\frac{n}{2}{\;\text{log}}_{10}\;\left(\frac{{\text{RSS}}_{\text{0}}}{{\text{RSS}}_{\text{1}}}\right)$$ (13)

where *n* is the sample size, RSS_0_ is the null residual sum of squares, and RSS_1_ is the residual sum of squares from the regression of the phenotype on the conditional QTL genotypes depending on markers genotypes. A permutation test with 1,000 replications was performed, repeatedly calling the ‘scanone’ function, for finding the threshold LOD scores for α = 0.05.

QTL analysis using the same methods was also performed on the observed data at maturity (final fruit DM and mean growth rate that were calculated as described in the genetic material and phenotypic data section) to validate the QTLs detected with the parameters of the growth models.

## Results

### Step 1: Unique solution optimized with nlme

**Fig 2:**
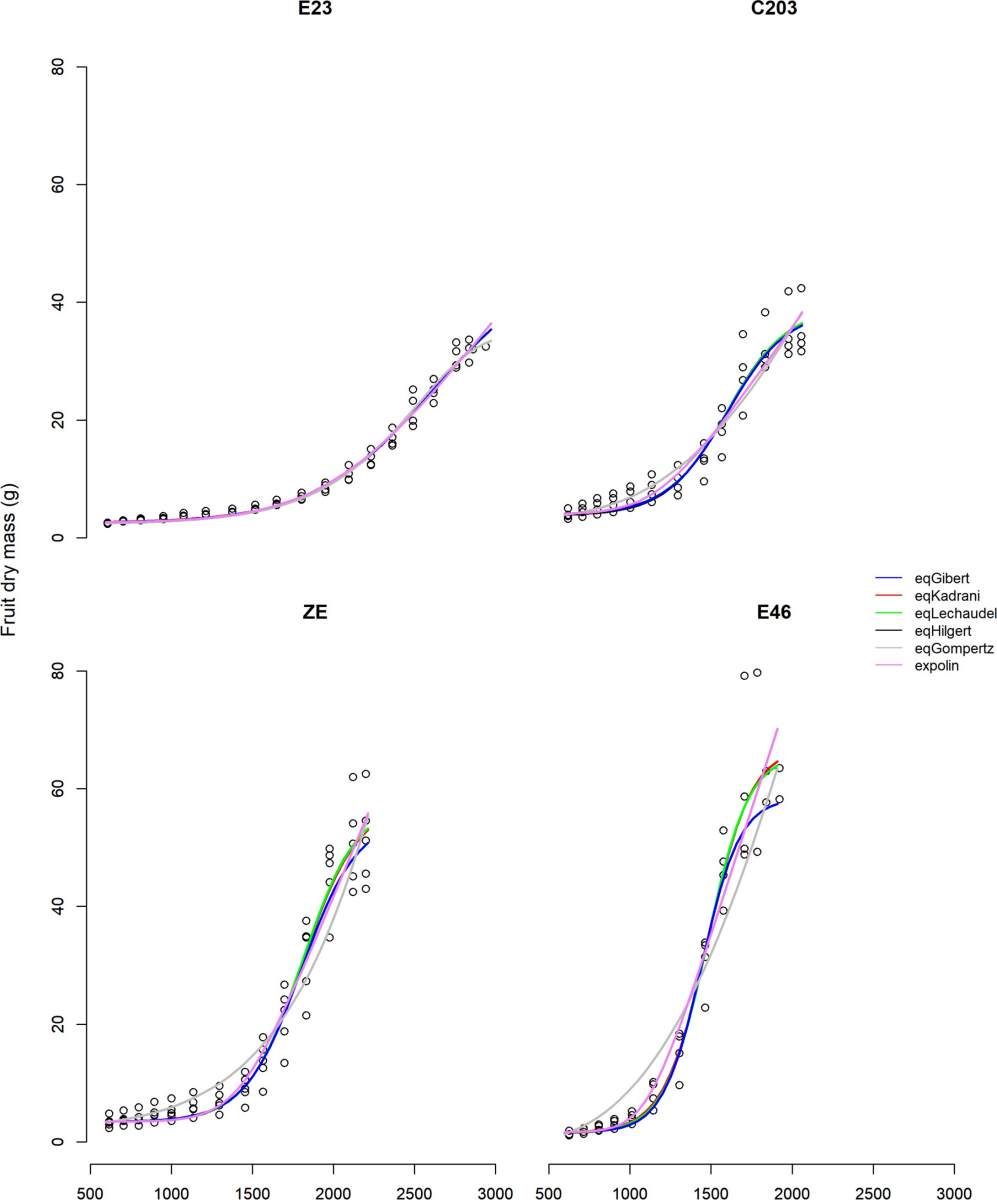
Observed fruit dry mass (g) and curves from 6 growth models through time in degree-days. Open black circles are the observed fruit dry mass measurements made during the study for genotypes E23, C203, ZE, E46. Curves of temporal development of growth were obtained with the unique solution estimated with the algorithm nlme for EqGibert (blue); eqKadrani (red); eqLechaudel (green); eqHilgert (black); eqGompertz (gray); and eqExpolin (pink).

The estimation of the parameters of the growth models obtained using the algorithm nlme failed for eqLogis.t, eqYin and eqRichards (nlme did not find a solution for these three growth models). The set of solutions obtained for the six remaining growth models (eqLogis.DM_max_ was not considered in Step1) was used in this first step to describe the temporal development of the growth curves and is shown in Fig 2 for four genotypes. For most of the genotypes, the growth models were superimposed and a good fit was observed for each (Figures A-D in S1 File).

The fit was significantly different for some equations, with the best and worst fits for eqExpolin and eqGompertz, respectively (Figure E in S1 File). No link was observed between the goodness of fit of the growth models and the number of parameters, but the introduction of parameter *B* seemed to improve the fit as seen for eqHilgert, eqKadrani and eqLechaudel compared to eqGibert (Figure E in S1 File).

**Fig 3:**
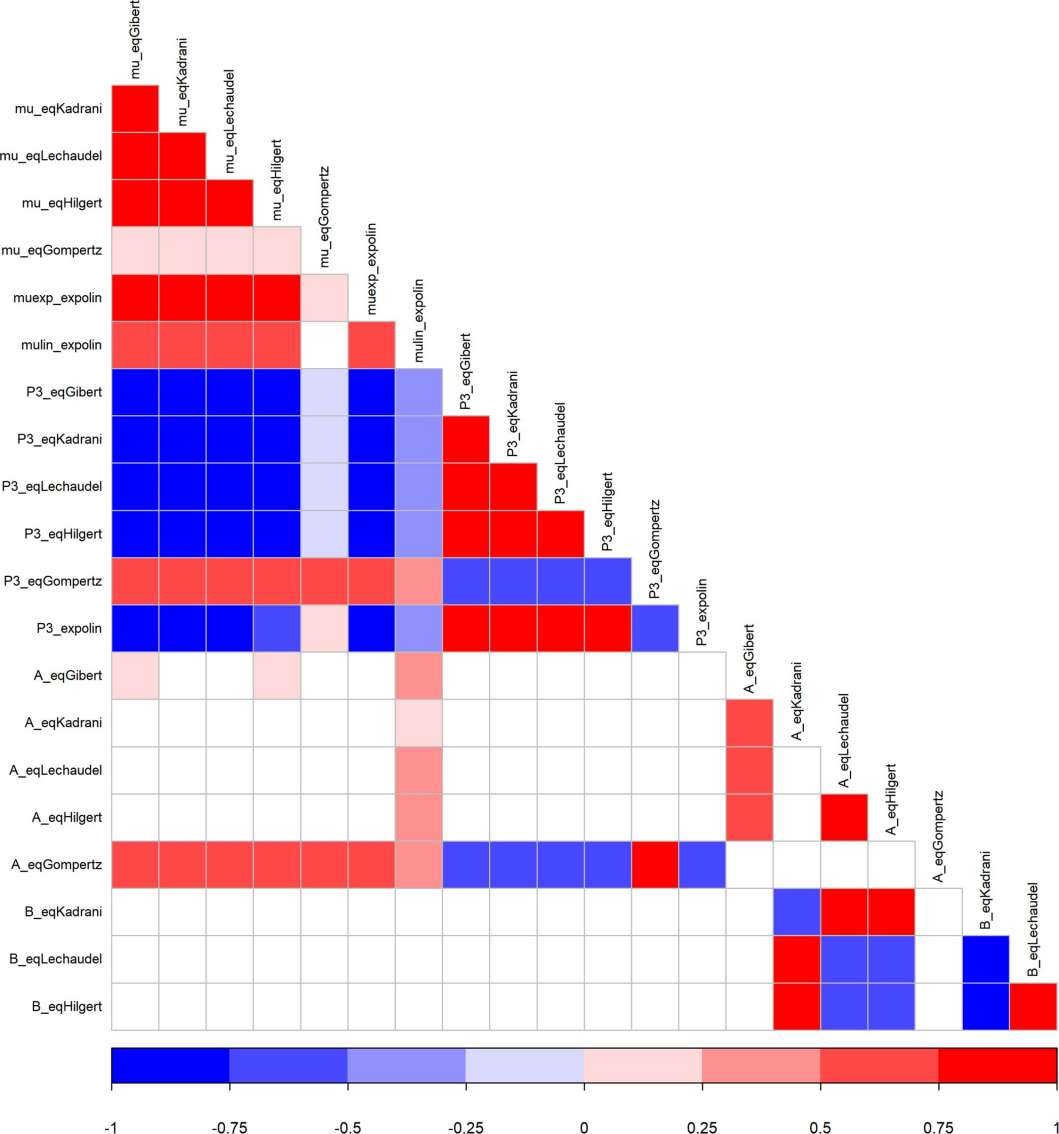
Heat map of correlations between and within parameters of 6 growth curves. Solutions of parameters obtained with the algorithm nlme in Step 1. Red shows positive correlations, blue shows negative correlations and white shows no correlation.

High correlations were observed between and within parameters of the growth models (Fig 3). Positive correlations were observed between parameters *mu* and *P3* in the different growth models (except *P3* of eqGompertz showed negative correlations with *P3* of the other growth equations). This result was expected as *mu* and *P3* have the same meaning in the growth equations used during the study. In addition, strong negative correlations were observed between parameters *mu* and *t*_*0*_ of eqExpolin and *mu* and *P3* of the other growth models (except for eqGompertz, where a positive correlation was observed). Parameter *A* of eqGompertz showed positive interactions with parameters *mu* and *P3* from the same growth model and a stronger interaction with the latter. Negative correlations were observed between *A* and *B* of eqHilgert and eqLechaudel (Fig 3) and positive correlations linked *A* and *B* parameters of eqKadrani with eqHilgert or eqLechaudel.

The parameters estimated with nlme were amenable to QTL analysis. No QTLs were detected with the parameters of eqGompertz, while the highest number of distinct QTLs was detected with eqExpolin (Table 1). QTLs were detected with *P3*, *t*_*0*_ and *mu* (including *mu*_*lin*_ and *mu*_*exp*_). No QTLs were detected for the *A* and *B* parameters. QTLs controlling *mu*, *mu*_*exp*_, *t*_*0*_ and *P3* were similar and only *mu*_*lin*_ displayed three specific QTLs (Table D in S1 File). In total, five distinct QTLs were detected with the parameters on linkage groups (LG): LG1.1; LG1.2; LG3.1; LG4.2; and LG6.2 (Table D in S1 File). No link was observed between the number of QTLs detected and the total number of parameters in the growth models. In contrast, the number of QTL detected was linked to the goodness of fit of the models, since the best and worst fits were observed for eqExpolin and eqGompertz, respectively (see Figure E in S1 File). The very closely related growth curves, derived from the logistic function [29], showed that different numbers of QTLs were detected with the parameters (Table 1), even when the equations counted the same number of parameters (see eqKadrani, eqLechaudel, and eqHilgert).

**Table 1:** Number of QTLs detected for the parameters of six growth models estimated with nlme (all parameters being genotype-dependent, Step 1).

			Nb QTL per parameter									
Equation	Nb* parameters	Nb* genotype- dependent parameters	*mu*	*P3*	*t*_*0*_	*A*	*B*	*mu*_*lin*_	*mu*_*exp*_	Nb* QTL	Nb* distinct QTL	Nb* major QTL
eqGibert	3	3	2	2	_	0	_	_	_	4	2	2
eqKadrani	4	4	2	2	_	0	0	_	_	4	2	2
eqLechaudel	4	4	1	2	_	0	0	_	_	3	2	2
eqHilgert	4	4	1	1	_	0	0	_	_	2	1	1
eqGompertz	3	3	0	0	_	0	_	_	_	0	0	0
eqExpolin	3	3	_	_	2	_	_	4	2	8	5	3

* Nb = number, _ = parameters not present in current growth model

To validate the QTLs detected with the parameters, QTL analysis was also performed on the observed data. QTLs detected with final fruit DM and mean growth rates calculated from the observed fruit data of the second back crossed population were found on LG1.1; LG1.2; LG4.2; LG5.1; and LG7.2 (Table 2). The growth model detecting the highest number of QTLs that were the same as those obtained from the observed data was eqExpolin (LG1.1; LG1.2; and LG4.2, see Table D in S1 File).

**Table 2:** QTLs detected with mean growth rate (meanGR) and final fruit DM (finalfruitDM) from observed data.

QTL	trait	LG	pos*	marker	LOD	p-value	effect	min	max
qtl_1.1_meanGR	meanGR	1	0	SNP_IGA_2272	3.11	0.01	6.02	0	6.5
qtl_1.1_DMmax	finalfruitDM	1	0	SNP_IGA_2272	2.72	0	8.28	0	16
qtl_1.2_meanGR	meanGR	1	31.1	FRU	2.99	0	7.88	28.1	44
qtl1.2_DMmax	finalfruitDM	1	31	FRU	3.80	0.004	4.56	28.1	47.4
qtl4.2_DMmax	finalfruitDM	4	18	c4.loc18	5.97	0	5.37	4	32
qtl4.2_meanGR	meanGR	4	31.4	CC3	7.01	0	6.49	16	34
qtl5.1_DMmax	finalfruitDM	5	14	c5.loc14	4.07	0	2.26	6.2	17
qtl5.1_meanGR	meanGR	5	14.6	SNP_IGA_561249	3.19	0.008	1.66	4	22
qtl7.2_DMmax	finalfruitDM	7	32	c7.loc32	3.75	0.004	1.41	26	37.9
qtl7.2_meanGR	meanGR	7	36	c7.loc36	2.81	0.026	0.41	28	41.2

* Pos = position

### Step 2: Genotype-independent parameters optimized with nlme

To deal with the high levels of correlation observed between the parameters of the equations, new sets of estimated parameters were produced using the algorithm nlme by defining from one to three parameters as genotype-independent (Table E in S1 File). No additional datasets could be produced for eqRichards. Some parameter values were aberrant for the unique dataset obtained for eqGompertz (Table E in S1 File). As in Step 1, the nlme algorithm failed to produce dataset solutions of the parameters for eqLogis.t.

**Fig 4:**
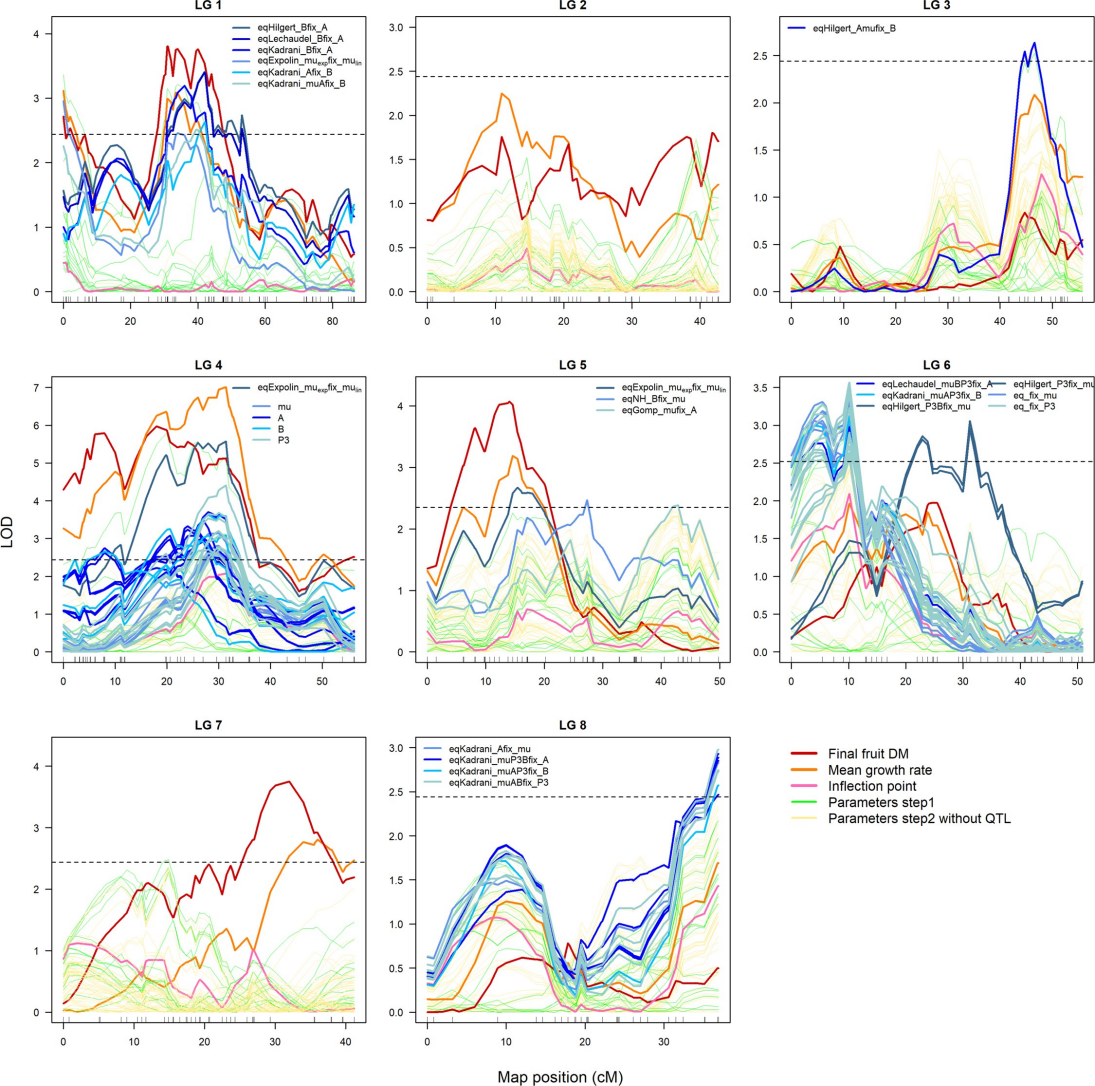
LOD profiles for the observed data and the parameters of the growth models estimated with nlme (Steps 1 and 2) on the 8 LGs. Observed data: final fruit dry mass (red); mean growth rate (orange); and inflection point (pink). LOD profiles for parameters estimated with nlme and all parameters being genotype-dependent are in green (Step 1). LOD profiles and QTL detected for parameters of the growth models when fixing some parameters as genotype-independent (Step 2) are in yellow (when no QTLs were detected) and shades of blue (depending on growth models, fixed parameters and parameters considered), respectively. When many QTLs were detected in the same LG, including when the same parameters and different growth models and/or fixed parameters, a generic label was used in the legend. The horizontal dashed lines represent the LOD thresholds used to detect QTLs.

Setting parameters as genotype-independent had the effect of significantly reducing the quality of the models fits, even if only moderately (Figure F in S1 File). Some growth models were more sensitive to this strategy. EqExpolin showed the best fit, while eqGompertz and eqYin showed the worst fit (Figure G in S1 File). Again, as in Step 1, eqGompertz was the growth model that allowed detection of the lowest number of QTLs (Table F in S1 File). For the other seven growth models (eqLogis.DM_max_ was again not considered in this step), QTLs were detected for *P3*, *t*_*0*_ and *mu* (including *mu*_*lin*_ and *mu*_*exp*_); they were also detected for *A* (with equations eqKadrani, eqLechaudel, eqHilgert, eqYin and eqGompertz) and *B* (with equations eqKadrani and eqHilgert) (Fig 4 and Table F in S1 File). The highest number of distinct QTLs was detected with the eqKadrani and eqHilgert growth models while setting only one parameter as being genotype-independent (Table F in S1 File). The results indicated no link between goodness of fit and the number of QTLs detected, as the best fit was observed with eqExpolin (Figure G in S1 File, See above). In general, a higher number of QTLs detected was observed by using this strategy (compared to using zero genotype-independent parameters, i.e., Step 1), with the exception of eqExpolin. Greater numbers of QTLs were detected by the growth curves derived from the logistic function [29] using four parameters (Table F in S1 File compared to the function using three parameters (eqGibert). The equations using four parameters (eqKadrani, eqLechaudel, eqHilgert) detected different numbers of QTLs depending on the number of parameters fixed as being genotype-independent (Table F in S1 File). Thus, as in Step 1, the very closely related growth curves showed different numbers of QTLs being detected.

By adopting this strategy, the major QTLs detected with the parameters of the growth models were the same (LG1.1, LG1.2, LG3.1, LG4.2 and LG6.2; see Fig 4) as the QTLs detected in Step 1 (zero genotype-independent parameters). Additional QTLs were detected with this strategy on LG4.1; LG5.1; LG5.3; LG5.4; LG6.1; LG6.3; and LG8.1 (Fig 4). Only QTL on LG7.2, detected with the observed data, was not detected with the parameters of the growth models when setting some parameters to being genotype-independent.

### Step 3: Parameters substituted with observed data and optimized with nlme

In this step, another strategy was adopted to deal with the high levels of correlations observed between the parameters of the growth models in Step 1. The strategy consisted of introducing ‘observed parameters’ to reduce the number of parameters to be estimated. In this manner, parameter *P3* was replaced by the average time of the inflection point calculated from the observed fruit data available per genotype for seven growth models (apart from equations eqLogis.t; eqExpolin; and eqLogis.DM_max_). In addition, eqLogis.DM_max_ was computed and compared to eqHilgert, as the former is a simplification of equation eqHilgert where *DMmax* is explicitly included and replaces parameters *A* and *B*. The latter strategy improved the goodness of fit (Figure H in S1 File) but did not improve the AIC value. Although the use of the observed inflection points did not significantly affect either the NMRSE or the AIC values, the general trend was the opposite. Slightly better results were obtained with the estimated *P3* compared to the *P3* from observed data (Figure I in S1 File).

When adopting this strategy, eqGibert, eqYin, eqGompertz and eqLogis.DM_max_ did not detect any QTL (Table G in S1 File). Similarly, no QTLs were detected for the equation eqLogis.DM_max_, despite good fitting results. Again, the results indicated no link between goodness of fit of a model and QTLs detected. Under this strategy the equation using three parameters (eqHilgert) detected more QTLs (five QTLs, Table G in S1 File) compared to the equations using four parameters (eqKadrani and eqLechaudel). The results indicated no link between the number of parameters used and the QTLs detected. In addition, eqKadrani detected three QTLs, while eqLechaudel detected four QTLs (Table G in S1 File). The results indicated that very closely related models, even with same number of parameters, can detect different QTLs.

**Fig 5:**
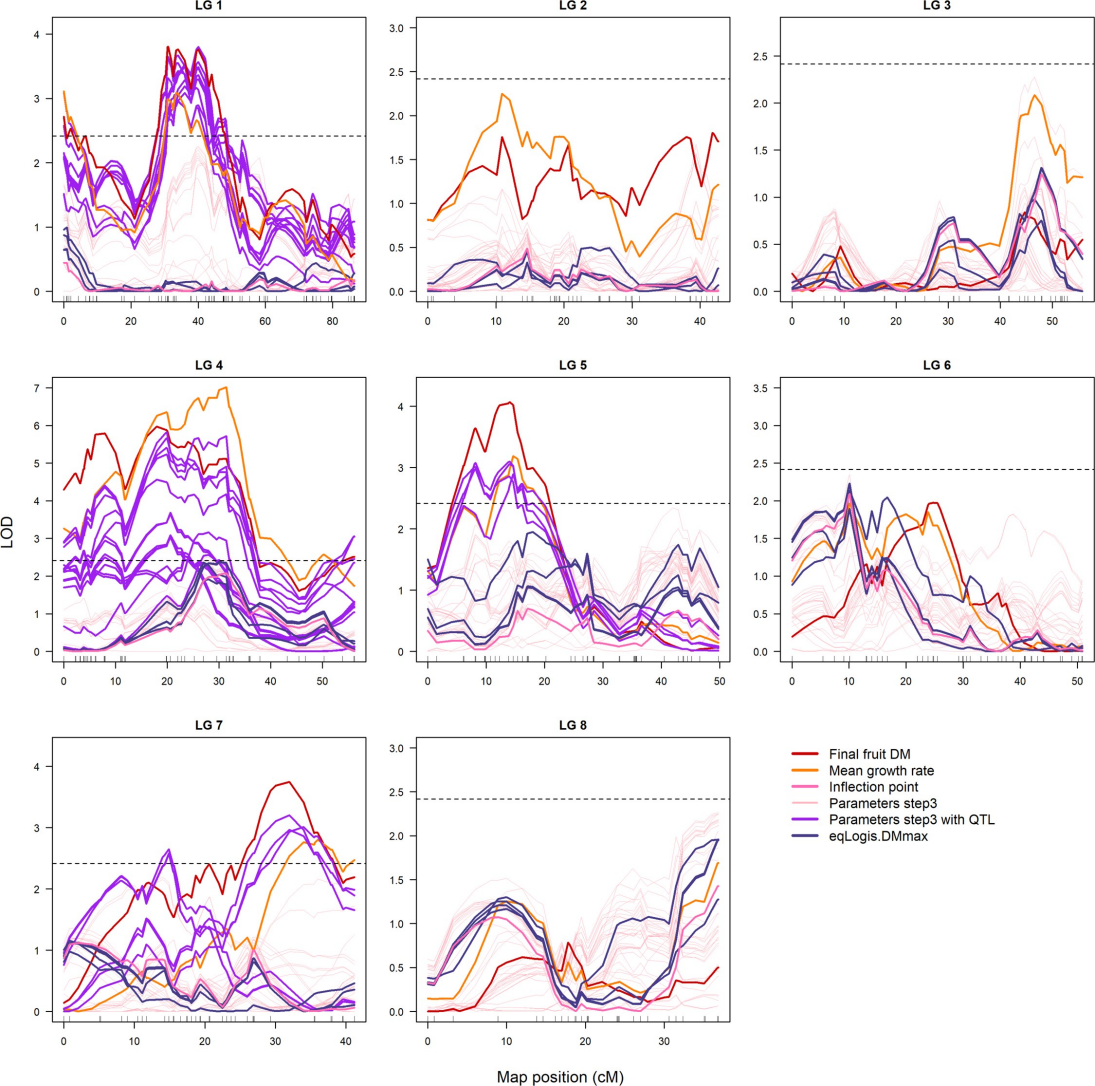
LOD profiles for the parameters of the growth models for the case that some parameters were substituted with observed data on the 8 LGs. LOD profiles and QTL detected for parameters of the growth models when replacing *P3* parameters by the average time of inflection points calculated from observed fruit data (Step 3) are in pale pink and purple, respectively. LOD profiles for parameters of equation eqLogis.DMmax, a simplification of Eq (6) where *DMmax* is explicitly included in place of the *A* and *B* parameters, are in color purple blue. Observed data: final fruit dry mass (red), mean growth rate (orange) and inflection point (pink) of the observed data. The horizontal dashed lines represent the LOD thresholds used to detect QTLs.

Under this strategy, QTLs were detected on LG1.1, LG1.2, LG4.1, LG4.2, LG5.1, LG7.1, and LG7.2 (Table G in S1 File). Parameter *P3* did not provide detection of any QTLs. Even if a lower number of QTLs was detected compared with the previous step (Step 2: use of genotype-independent parameters), this strategy allowed the detection of the same QTLs as were obtained with observed data (Fig 5).

### Step 4: Multiple solutions optimized with the RBGA algorithm

To overcome some drawbacks of classical algorithms such as nlme, the genetic algorithm RBGA was used in this step. As with all genetic algorithms, RBGA is a population-based algorithm and produces at the end of each run a set of best solutions found. RBGA was run 20 times for each growth model and the best solution found for each run was selected and collected, along with the two extreme solutions (see multiple-solutions selected in parameters estimation section), into a dataset. When comparing the best solution obtained with RBGA and the unique solution obtained with nlme, the parameter values of some genotypes were quite different (Figure J in S1 File). A strong effect of the algorithm was observed on the goodness of fit and AIC, with best fit occurring when using the best solutions from RBGA and the worst fit took place with the unique solution from nlme (Figure K in S1 File). This was true for all six (results of nlme) growth models being compared (Figure L in S1 File).

**Fig 6:**
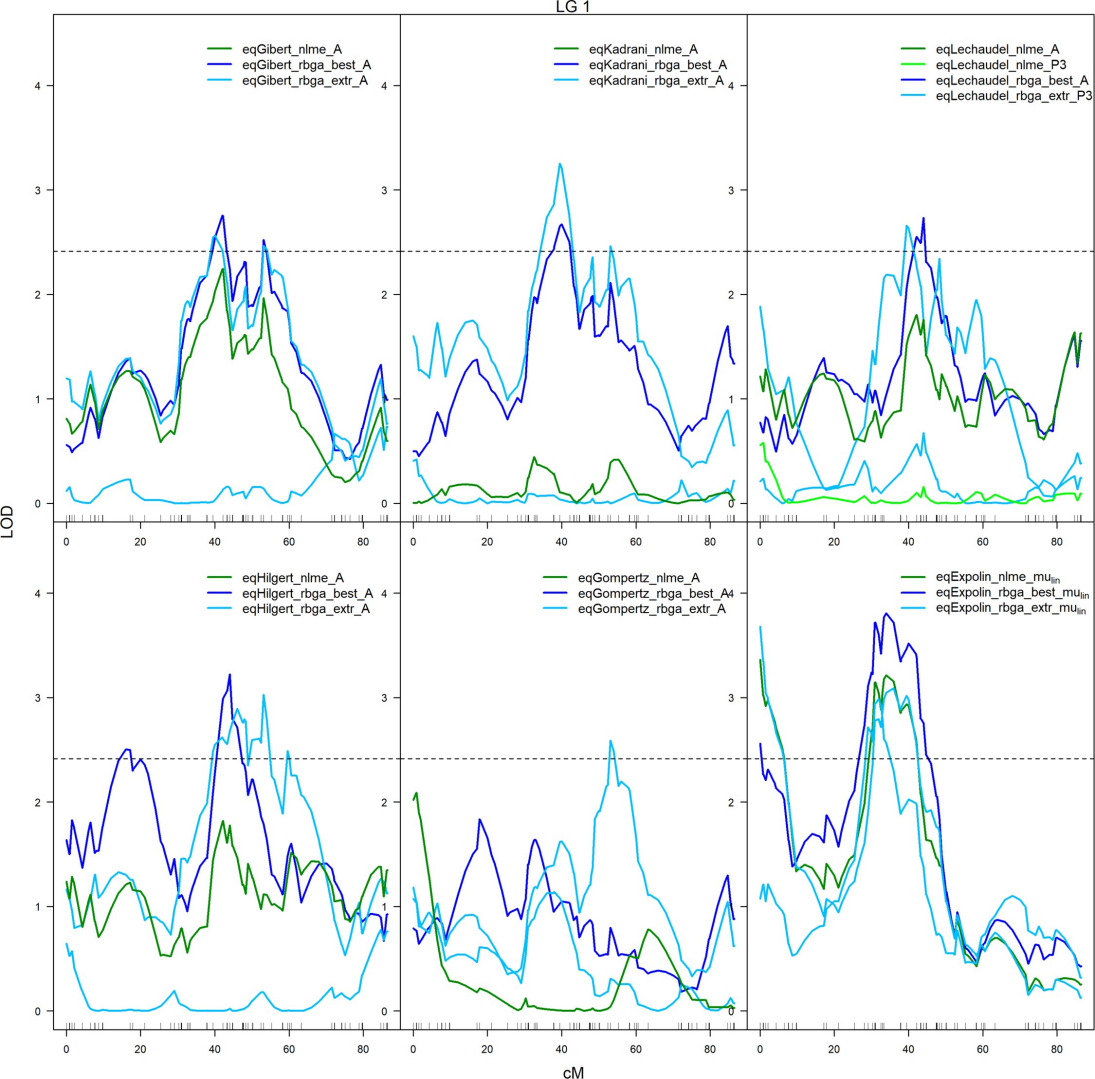
QTLs detected on LG1 for the parameters estimated with RBGA and the corresponding LOD profiles for same parameters estimated with nlme for 6 of the growth models: EqGibert, eqKadrani, eqLechaudel, eqHilgert, eqGompertz, eqExpolin. The solutions ‘best’ and ‘extr’ refer respectively to the best solution (navy blue) and the extreme solutions (light blue) estimated with RBGA. In green, LOD profiles for parameters estimated with nlme (results from Step 1 with genotype-dependent parameters only) for which QTL were detected with RBGA estimation. The horizontal dashed lines represent the LOD thresholds for detecting QTLs.

The variability in parameter values between the solutions obtained with nlme and with RBGA led to variation in the QTL profiles. In most cases, QTL locations and peak shapes were very comparable between solutions (Fig 6). However, it is significant that QTLs were not detected or were detected at quite different locations. This was observed, for example, with eqHilgert since the QTL obtained on LG1 with extreme RBGA solutions had a different location than the QTL detected with the best RBGA and with the unique nlme solution (Fig 6). The best solution from RBGA did not always result in the best LOD score, as was found in Fig 6 for eqKadrani. The best and the extreme solutions from RBGA generally detected the same number of distinct QTLs with slightly more QTLs detected using the best solutions (Table H in S1 File). The growth model detecting the largest number of distinct QTLs (when considering the best and extreme solutions) was eqLogis.t, while the smallest number of QTLs was detected with eqYin, eqGompertz and eqLogis.DM_max_ (Table H in S1 File). The QTLs were detected for parameters *A*, *P3*, *mu* (including *mu*_*lin*_ and *mu*_*exp*_), *t*_*0*,_ *TE* and *V*.

As in Steps 1–3, the very closely related growth curves showed different numbers of QTLs being detected. Under this strategy, the equations using three parameters (eqHilgert and eqLogis.t) detected more QTLs (Table H in S1 File) compared to the equations with four parameters (eqKadrani and eqLechaudel). These results indicated no link between the number of parameters and the QTLs detected. EqLogis.t detected 8 QTLs, while eqHilgert detected four QTLs (Table H in S1 File). The results indicated that very closely related models, even with same number of parameters to be estimated, can detect different QTLs.

Under this strategy, QTLs were detected on LG1.1; LG1.2; LG1.3; LG4.1; LG4.2; LG5.1; LG5.2; LG6.1; LG6.2; LG7.2; and LG8.1 (Table H in S1 File). This strategy (Step 4) detected all QTLs detected with the observed data (Table 2) and same high number of QTLs detected under Step 2 (Table H in S1 File).

When comparing all strategies used in this study, the growth model that detected the largest number of distinct QTLs was eqHilgert, while the smallest number of QTLs was detected with eqLogis.DM_max_ (Table H in S1 File). A total of 3 major QTLs was detected in numerous cases. Two of the major QTLs, on LG1 and LG4, were detected both with observed data and with estimated parameters (Table H in S1 File). In addition, other QTLs were detected on LG1, LG5 and LG7 with observed data but were rarely detected with estimated parameters. The growth models detecting those QTLs on LG1, LG5 and LG7 were eqLogis.t, eqExpolin, eqHilgert, eqLogis.DM_max_ (Table H in S1 File). Furthermore, 10 other QTLs were detected only with estimated parameters and, among these, two were frequently detected (LG4.1 and LG8.1). Both LG4.1 and LG8.1 were detected with eqLogis.t, eqKadrani, eqLechaudel and eqHilgert. In addition, LG 4.1 was also detected with eqLogis.t, and LG8.1 was also detected with eqGibert, eqYin and eqRichards (Table H in S1 File).

## Discussion

The case study described in this research has attempted to systematically investigate the effect of different growth curves (that are closely and not closely related to each other, and characterized by large and small numbers of parameters) on the detection of the QTLs that control the parameters capturing the biological process of peach growth. The ultimate goal of this study is to improve the future QTL-based ecophysiological modeling approach.

First, the results of this study are dependent on several factors directly linked to the efficiency of the QTL analysis, such as the size of the genetic population, and the density and resolution of the linkage map. The genetic map used in this study is fairly accurate compared to the maps usually used for genetic mapping studies in *Prunus* [47]. Therefore, the limiting factor impairing QTL detection and resolution in this study might be the number of genotypes studied. However, in *Prunus* species, single seed production per fruit and juvenile period constraints result in rather small number of genotypes studied compared to those typically used for genetic mapping studies [47]. The range extends from 48 to 270 descendants in peach progenies, with a median of 77. Consequently, we believe that the study proposed here including 161 genotypes is reasonable. In addition, the BC2 population studied displays a very large range of variation in traits [48], which makes it of particular interest, even with a limited number of individuals. Generally, the larger the size of the genetic population is, the higher the power of QTL detection is [49–51]. Further, the positions of QTLs can be slightly different, and the confidence intervals may be reduced. The effect of QTLs is often overestimated. Nevertheless, the present study focuses on the approximate position of major QTLs to compare growth models fitted from exactly the same data and number of genotypes. Thus, all the results suffer from the same limitation. In consequence, we believe that the results obtained from the QTL analyses are suitable for reaching our goal.

What was expected during the QTL analysis was results showing that the same QTLs were detected with the observed data and the estimated parameters of the growth models, but the estimated parameters were expected to detect more QTLs since parameters can decipher the different phases and processes involved in fruit DM development. Indeed, the underlying postulate of this work is that the capacity to detect a ‘real’ QTL results from how the model parameters (or sets of correlated parameters) capture the biological processes controlled by genes. The best QTL results may come from the best representation of the biological processes by the models and/or model parameters tightly linked to biological processes controlled by genes.

The growth models were able to detect, with sufficient frequency to be robust, new QTLs undetected with the observed data. However, the results were quite different between the growth models and dependent on the strategies used. The number of QTLs detected fluctuated between the growth models and strategies, but they mostly showed similar LOD profiles and generally differed in the heights of the peaks and whether or not the peaks reached significance. In the first step, eqExpolin detected the highest number of distinct QTLs. When using the strategy of the second step, eqKadrani and eqHilgert detected the highest numbers of distinct QTLs. Finally, when optimizing the parameters with RBGA, eqLogis.t detected the highest number of distinct QTLs.

To the best of our knowledge, the study by Wu et al. [18] was the only scientific work that analyzed the impact of different growth models on the QTL detection. In contrast to what Wu et al. [18] found, in our study the number of QTLs detected did not relate to the goodness of fit of the parameterized growth models. Wu et al. [18] used a multiple-trait approach that better considered correlations between traits, showing that QTL detection based on analysis of growth curve models gives similar QTLs provided that the chosen growth functions fit the data satisfactorily. Although this is partly true for the results obtained in Step 1, we could not generalize this to all of our results. Using the strategy adopted in the second step, the best fit was observed with eqExpolin, but the highest number of distinct QTLs was obtained with the two growth models indicated above. With the strategy adopted in the third step, we observed better fitting with eqLogis.DM_max_ than with eqHilgert, but the latter detected more QTLs among which some were the main ones. However, since the range of goodness of fit explored in our study was not very large, it is not appropriate to generalize these results. Models for which the goodness of fit is lower than that observed in this study would probably not allow detection of true QTLs. The very high sensitivity of the QTL detection to the variations in solutions that we observed in this study suggests that the QTL detection of model parameters with poor goodness of fit would lead to a deadlock. In addition our study went further than the research study by Wu et al. [18], because it analyzed the impacts of very closely related growth models on QTL detection. Further we found (for all steps used during the study, Steps 1–4) that even closely related growth models (using the same number of parameters) can lead to detection of different QTLs.

Finally, the detection of QTLs was not linked to the complexity of the growth models, since the equations with 3 parameters (eqExpolin and eqLogis.t), and not with 4 parameters, detected the highest number of QTLs when using both nlme (results from Step 1 only) and RBGA. The findings indicated uncertainties in using the goodness of fit and the complexity of a growth model to select a model for the QTL-based ecophysiological model approach. Thus, all findings highlighted that testing of different growth models is an important step to be performed before building a QTL-based ecophysiological model.

Our study also attempted to indirectly investigate the effect of correlations between parameters on the detection of QTLs, since the bottom-up approach used does not allow correlation analysis in the parameterization process. High correlations were observed between the parameters of each growth model resulting in interchanging sets of solutions and thus in the difficulty of estimating the ‘true’ genetic value of the parameters. This ‘floating’ between possible values of correlated parameters is illustrated by the variability of good solutions found by the RBGA algorithm with each having very good evaluation values. Depending on the choice of the solution for each genotype, this can lead to a rank inversion of the genotypes for their parameter values, and thus lead to different QTLs being detected. To deal with these correlations, two different strategies were adopted. The first one consisted on considering one to three parameters as being genotype-independent (Step 2 in the study), and thus concentrating the entire genetic variability on the remaining parameters to be estimated. The second strategy was based on the replacement of parameters *P3*, *A* and *B* with observed data (Step 3 in the study). The two strategies caused slight reductions in goodness of fit (unless the test with *DMmax* is explicitly included. See results of Step 3 compared to those of Step 1) and resulted in detecting different QTLs compared to those detected in Step 1. The first strategy (Step 2) detected more QTLs than were detected in Step 1. However, as shown in the results, a QTL on LG7.2 (detected with observed data) was not observed. The second strategy (Step 3) detected very few QTLs, but same as the ones detected with the observed data. These results suggest that calculating one parameter independently from observed data (as was done for *P3* in Step 3) before estimating the other parameters may not be a good strategy. When a parameter is directly observed (the case of *DMmax* in Step 3), the results seem to show improvement. Finally, the strategy of setting parameters independent of the genotype may be recommended. Given the uncertainties in the solutions, it was important to have the QTLs detected with observed data as a reference.

Concerning the algorithms, as expected, the best solution obtained with RBGA showed better fitting than the unique solution obtained with nlme, which also did not allow fitting some equations such as eqLogis.t. The latter is a classical algorithm which can get ‘stuck’ at a local minimum, without having the possibility of exploring other solutions spaces. In some cases, it failed to find a solution, or some of the values obtained were aberrant. Since RBGA is an evolutionary algorithm and it is based on stochasticity, there is a higher probability of finding the global minimum and therefore solutions with better fitting to the growth models. As observed in the results, the variability in the parameter values led to variations in the QTL profiles. Together with the correlation between parameters, the results showed the issue of obtaining the ‘true’ genetic parameters for the improvement of the QTL-based approach. The strategy with genotype-dependent parameters and parameters replaced with observed data, as tested with nlme in our study, has undoubtedly suffered from the inherent and well-known limitations of the nlme classical algorithm. Although the RBGA algorithm showed better performance than nlme, both algorithms showed poor performance when addressing the research question. Even if we had only three or four parameters to estimate, when dealing with constraints linked to genotype-dependent parameters, the dimensions of the problem becomes high due to the number of individuals (genotypes) considered. For instance, let us consider a model having three parameters to be fitted (one genotype-independent parameter and two genotype-dependent parameters). The total number of decision variables to be optimized at the same time in our genetic peach population (161 genotypes) will be at least (2*161+1 = 323) with some simplifications. Thus, the resulting optimization problem involves a high number of variables (parameters in our case). Those problems involving more than 100 variables are known as large-scale global optimization problems (LSGO). Although, metaheuristics are powerful for addressing some difficult optimization problems, these algorithms show poor performance when dealing with the curse of dimensionality, i.e., LSGO. The curse of dimensionality refers to the exponential growth of the search space when the number of decision variables grows linearly. This issue has a high impact on the performance of many well-known metaheuristics (as discussed by Chen et al. [52]) for particle swarm optimization and differential evolution. We arrived at the same conclusions when we attempted without success (results not shown) to use a differential evolution algorithm available within the framework of the DEoptim R package. One of the main limitations of existing global search algorithms is their lack of scalability for tackling the curse of dimensionality. This is why, from our point of view, RBGA and DEoptim struggle to fit our models in a satisfactory way.

A growing interest in developing more effective algorithms to deal with LSGO problems has been observed in recent years. However, from our point of view, this development is still in the beginning stages, especially regarding black-box constrained LSGO problems [53]. A recent review of these developments has been published by Mahdavi et al. [54]. Therefore, new solutions should be discussed by the scientific community to develop new algorithms suitable for LGSO to better address the problem of correlations between parameters (as done by adopting Strategy 2 in our study), especially for fitting crop models involving a large number of parameters.

Additional studies are needed to confirm the results of this work. Despite the fact that it is premature to generalize rules for how to best select a model for the QTL-based ecophysiological modeling approach, initial conclusions can be drawn from this study. The importance of this scientific work lies in the exploration of other solution spaces for the parameters and in pointing out the importance of algorithm improvement if the scientific community is interested in the use of the bottom-up approach for QTL-based ecophysiological modeling.

## Supporting information

S1 File **Table A.** Parameters of the ten growth model equations. **Table B.** Genotype-independent parameters for the second step strategy. **Table C.** GA settings during the growth model parameterizations. **Figures A-D.** Observed fruit dry mass (g) and curves from 9 growth models along time in degree-days. **Figure E.** Variability of NRMSE (above) and AIC (below) in the genetic population obtained for 6 growth models with nlme algorithm, all parameters being genotype-dependent (Step 1). **Table D.** QTLs detected for the parameters of six growth models estimated with nlme (Step 1). **Table E.** Successful calibrations when fixing from 1 to 3 parameters as genotype-independent with nlme for 8 growth models. **Figure F.** Variability of NRMSE (left) and AIC (right) in the genetic population obtained with nlme algorithm by fixing from zero to three parameters as genotype-independent (Step 2). **Figure G.** Variability of NRMSE (left) and AIC (right) in the genetic population obtained with nlme algorithm by fixing from zero to three parameters as genotype-independent (constant) for 8 growth models (Step 2). **Table F.** Number of QTLs detected with the parameters of the growth models estimated with nlme by fixing from 1 to 3 parameters as genotype-independent. **Figure H.** Effect of replacing parameters by observed data on the variability of NRMSE (left) and AIC (right) in the genetic population obtained by using the nlme algorithm. **Figure I.** Effect of replacing P3 parameter by observed data on NRMSE and AIC values. (A) Correlation between NRMSE (upper-left) and AIC (upper-right) values and (B) variability of NRMSE (bottom-left) and AIC (bottom-right) calculated with observed and optimized P3 by using the nlme algorithm for 6 growth models (Step 3). **Table G.** QTLs detected with parameter solutions of nine growth models obtained by replacing the parameters with average inflection point and final dry mass with nlme. **Figure J.** Representation per genotype of the values of parameters mu and A obtained per genotype with RBGA and nlme algorithms using eqHilgert growth model. **Figure K.** Comparison of NMRSE and AIC for solutions obtained with nlme and RBGA algorithms for 6 growth models (Step 4). **Figure L.** Variability of NRMSE (above) and AIC (below) for solutions obtained with nlme (uniques) and RBGA (Best, Extremes1 and Extremes2) algorithms for 6 growth models (Step 4). **Table H.** QTLs detected with parameter solutions of ten growth models obtained by parameterizations during the study with nlme and RBGA.
(DOCX)

S1 Data " FruitGrowthData_progeny.csv " contains observed data of fruit growth as CSV file. (CSV)

S2 Data " GeneticMap_GenotypeData.xlsx" contains molecular markers data and genetic map data as XLS file. (XLSX)
