# Supplementary material for: Model-based QTL detection is sensitive to slight modifications in model formulation
Source: PLoS One. 2019 Oct 3;14(10):e0222764. doi: 10.1371/journal.pone.0222764 (PMC6776317; doi:10.1371/journal.pone.0222764)
Supplement: S1 File — Table A. Parameters of the ten growth model equations. Table B. Genotype-independent parameters for the second step strategy. Table C. GA settings during the growth model parameterizations. Figures A-D. Observed fruit dry mass (g) and curves from 9 growth models along time in degree-days. Figure E. Variability of NRMSE (above) and AIC (below) in the genetic population obtained for 6 growth models with nlme algorithm, all parameters being genotype-dependent (Step 1). Table D. QTLs detected for the parameters of six growth models estimated with nlme (Step 1). Table E. Successful calibrations when fixing from 1 to 3 parameters as genotype-independent with nlme for 8 growth models. Figure F. Variability of NRMSE (left) and AIC (right) in the genetic population obtained with nlme algorithm by fixing from zero to three parameters as genotype-independent (Step 2). Figure G. Variability of NRMSE (left) and AIC (right) in the genetic population obtained with nlme algorithm by fixing from zero to three parameters as genotype-independent (constant) for 8 growth models (Step 2). Table F. Number of QTLs detected with the parameters of the growth models estimated with nlme by fixing from 1 to 3 parameters as genotype-independent. Figure H. Effect of replacing parameters by observed data on the variability of NRMSE (left) and AIC (right) in the genetic population obtained by using the nlme algorithm. Figure I. Effect of replacing P3 parameter by observed data on NRMSE and AIC values. (A) Correlation between NRMSE (upper-left) and AIC (upper-right) values and (B) variability of NRMSE (bottom-left) and AIC (bottom-right) calculated with observed and optimized P3 by using the nlme algorithm for 6 growth models (Step 3). Table G. QTLs detected with parameter solutions of nine growth models obtained by replacing the parameters with average inflection point and final dry mass with nlme. Figure J. Representation per genotype of the values of parameters mu and A obtained per genotype with [file pone.0222764.s001.docx]

**S1 File. Table A.** **Parameters of the ten growth model equations**

| **Symbol** | **Meaning** |
| --- | --- |
| *A* | Parameter involved in the calculation of the sigmoid curve asymptote estimating *DM_max_* [dimensionless] |
| *B* | Parameter involved in the calculation of the sigmoid curve asymptote estimating *DM_max_* [g] or [dimensionless]* |
| *mu* | Initial relative fruit growth rate [GDD^-1^] |
| *mu_lin_* | Maximum growth rate in the initial phase [g GDD^-1^] |
| *mu_exp_* | Maximum growth rate in the exponential phase [GDD^-1^] |
| *P3* | Time of the inflexion point of the fruit growth curve [GDD] |
| *TE* | Time at the end of the growth period [GDD] |
| *t_0_* | Time at which the linear phase effectively begins [GDD] |
| *V* | Parameter involved in asymmetrical growth [dimensionless] |

*[dimensionless] for equations (3) and (5)

**S1 File. Table B.** **Genotype-independent parameters for the second step strategy**

|  |  | **Constant parameters** | | |
| --- | --- | --- | --- | --- |
| **Equation number** | **Parameters** | **1** | **2** | **3** |
| (1) | *A, mu, P3* | *mu* | [*A, mu*] | *-* |
| (2) | *A, B, mu, P3* | *A, B, mu* | [*A, mu*] | [*A, B, mu*], [*A, P3, mu*] |
| (3) | *A, B, mu, P3* | *B, mu* | [*B, mu*] | [*B, P3, mu*] |
| (5) | *A, B, mu, P3* | *B, P3, mu* | [*A, mu*], [*B, P3*], [*B, mu*] | [*A, B, mu*], [*B, P3, mu*] |
| (6) | *A, TE, P3* | *A, TE, P3* | [*A*, *TE*] | *-* |
| (7) | *A, V, mu, P3* | *V* | - | *-* |
| (8) | *A, mu, P3* | *mu* | - | *-* |
| (9) | *mu_lin_, mu_exp_, t_0_* | *mu_lin_, mu_exp_* | [*mu_lin_, mu_exp_*] | *-* |

**S1 File. Table C.** **GA settings during the growth models’ parameterizations**

| Population size | 100 |
| --- | --- |
| Maximum number of iterations | 100 |
| Mutation chance | 0.1 |
| Elitism | 2 |

 **S1 File. Figures A-D.** **Observed fruit dry mass (g) and curves from 9 growth models along time in degree-days.** Open black circles are observed fruit dry mass available during the study for each genotype. Curves of temporal development of growth were obtained with the unique solution estimated with the algorithm *nlme*. Exception are eqYin and eqRichards, because the observed temporal development was obtained by fixing to constant value respectively the parameters *A* and *V* respectively. EqHilgert (black), eqKadrani (red), eqLechaudel (green), eqGibert (blue), eqGompertz (grey), eqExpolin (pink), eqlogis.DM_max_ (brown), eqYin_Afix (turquoise dashed line), eqRichards_Vfix (orange dashed line).

**S1 File. Figure E.** **Variability of NRMSE (above) and AIC (below) in the genetic population obtained for 6 growth models with *nlme* algorithm, all parameters being genotype-dependent** **(step 1).** The band inside the box represents the median. The ends of the dashed lines represent the lowest and the highest values still within the 1.5 inter-quartile range of the respectively lower and upper quartile. Circles represent genotypes outliers. One-way ANOVA was performed. Significant growth model effect was observed on NRMSE (*p* = 7.38E^-07^) and AIC (*p* = 0.000406).

**S1 File. Table D.** **QTLs detected for the parameters of six growth models estimated with *nlme* (step 1)**

| **QTL** | **Equation** | **Trait** | **Nb**  **parameters**  **estimated** | **LG** | **Position (cM)** | **Marker** | **LOD** | **p-value** | **Effect** | **min** | **max** |
| --- | --- | --- | --- | --- | --- | --- | --- | --- | --- | --- | --- |
| qtl_1.1_mu | eqExpolin | mu_lin_ | 3 | 1 | 0 | SNP_IGA_2272 | 3.36 | 0.002 | 3.71 | 0 | 7.6 |
| qtl_1.2_mu | eqExpolin | mu_lin_ | 3 | 1 | 34.0 | c1.loc34 | 3.21 | 0.0002 | 8.02 | 28.1 | 43.2 |
| qtl_3.1_mu | eqExpolin | mu_lin_ | 3 | 3 | 46.6 | SNP_IGA_358726 | 2.52 | 0.039 | 5.00 | 42 | 52.9 |
| qtl_4.2_mu | eqExpolin | mu_lin_ | 3 | 4 | 19.9 | SNP_IGA_387459 | 5.77 | 0 | 10.17 | 14 | 32 |
| qtl_4.2_mu | eqGibert | mu | 3 | 4 | 29.6 | SNP_IGA_399858 | 2.51 | 0.05 | 3.81 | 24 | 35.8 |
| qtl_4.2_P3 | eqGibert | P3 | 3 | 4 | 30 | c4.loc30 | 2.54 | 0.045 | 3.77 | 25.2 | 35.8 |
| qtl_4.2_mu | eqExpolin | mu_exp_ | 3 | 4 | 30 | c4.loc30 | 3.13 | 0.012 | 5.55 | 23.4 | 35.8 |
| qtl_4.2_mu | eqKadranirani | mu | 4 | 4 | 30 | c4.loc30 | 2.59 | 0.043 | 4.15 | 24 | 35.8 |
| qtl_4.2_P3 | eqKadranirani | P3 | 4 | 4 | 30 | c4.loc30 | 2.74 | 0.035 | 4.28 | 25.2 | 35.8 |
| qtl_4.2_P3 | eqLechaudel | P3 | 4 | 4 | 30 | c4.loc30 | 2.63 | 0.033 | 4.11 | 25.2 | 35.8 |
| qtl_4.2_t_0_ | eqExpolin | t_0_ | 3 | 4 | 31.4 | CC3 | 2.44 | 0.045 | 4.25 | 25.2 | 35.8 |
| qtl_6.2_mu | eqGibert | mu | 3 | 6 | 10.1 | SNP_IGA_619845 | 3.34 | 0.005 | 6.64 | 0 | 12 |
| qtl_6.2_P3 | eqGibert | P3 | 3 | 6 | 10.1 | SNP_IGA_619845 | 3.36 | 0.006 | 6.21 | 0 | 12 |
| qtl_6.2_mu | eqExpolin | mu_exp_ | 3 | 6 | 10.1 | SNP_IGA_619845 | 3.31 | 0.006 | 5.85 | 0 | 12 |
| qtl_6.2_t_0_ | eqExpolin | t_0_ | 3 | 6 | 10.1 | SNP_IGA_619845 | 2.97 | 0.011 | 5.33 | 0 | 13 |
| qtl_6.2_mu | eqKadranirani | mu | 4 | 6 | 10.1 | SNP_IGA_619845 | 3.33 | 0.006 | 6.52 | 0 | 12 |
| qtl_6.2_P3 | eqKadranirani | P3 | 4 | 6 | 10.1 | SNP_IGA_619845 | 3.43 | 0.005 | 5.85 | 0 | 12 |
| qtl_6.2_mu | eqLechaudel | mu | 4 | 6 | 10.1 | SNP_IGA_619845 | 3.42 | 0.005 | 9.74 | 0 | 12 |
| qtl_6.2_P3 | eqLechaudel | P3 | 4 | 6 | 10.1 | SNP_IGA_619845 | 3.47 | 0.002 | 6.21 | 0 | 12 |
| qtl_6.2_mu | eqHilgert | mu | 4 | 6 | 10.1 | SNP_IGA_619845 | 2.61 | 0.043 | 7.79 | 0 | 13 |
| qtl_6.2_P3 | eqHilgert | P3 | 4 | 6 | 10.1 | SNP_IGA_619845 | 3.14 | 0.008 | 7.84 | 0 | 12 |

**S1 File. Table E.** **Successful calibrations when fixing from 1 to 3 parameters as genotype-independent with *nlme* for 8 growth models**

| **Equation number** | **Name of growth model** | **Parameters** | **1 as constant** | **2 as constants** | **3 as constants** | **Nb of datasets** | **Total nb of genotypic series of parameters** |
| --- | --- | --- | --- | --- | --- | --- | --- |
| (1) | eqGibert | *A*, *mu*, *P_3_* | *mu* | [*A*, *mu*] | - | 2 | 3 |
| (2) | eqKadrani | *A*, *B*, *mu*, *P_3_* | *A*, *B*, *mu* | [*A*, *mu*] | [*A*, *B*, *mu*], [*A*, *P_3_*, *mu*] | 6 | 13 |
| (3) | eqLechaudel | *A*, *B*, *mu*, *P_3_* | *B*, *mu* | [*B*, *mu*] | [*B*, *P_3_*, *mu*] | 4 | 9 |
| (5) | eqHilgert | *A*, *B*, *mu*, *P_3_* | *B*, *P_3_*, *mu* | [*A*, *mu*], [*B*, *P_3_*], [*B*, *mu*] | [*A*, *B*, *mu*], [*B*, *P_3_*, *mu*] | 8 | 17 |
| (6) | eqYin | *A*, *TE*, *P_3_* | *A*, *TE*, *P_3_* | [*A*, *TE*] | - | 4 | 7 |
| (7) | eqRichards | *A*, *V*, *mu*, *P_3_* | *V* | - | - | 1 | 3 |
| (8) | eqGompertz | *A*, *mu*, *P_3_* | *mu* | - | - | 1^$^ | 2 |
| (9) | eqExpolin | *mu_lin_*, *mu_exp_*, *t_0_* | *mu_exp_* | [*mu_exp_*, *mu_lin_*] | - | 2 | 3 |

^$^some parameter values were aberrant

**S1 File. Figure F.** **Variability of NRMSE (left) and AIC (right) in the genetic population obtained with *nlme* algorithm by fixing from zero to three parameters as genotype-independent (step 2).** The eight growth models included in the analysis are: eqGibert, eqKadrani, eqLechaudel, eqHilgert, eqYin, eqRichards, eqGompertz, eqExpolin. The ends of the dashed lines represent the lowest and the highest values still within the 1.5 inter-quartile range of the respectively lower and upper quartile. Circles represent genotypes outliers. One-way ANOVA was performed. Significant strategy effect was observed on NRMSE (*p* = 3.1e^-09^) and AIC (*p* = 5.38e^-06^).

**S1 File. Figure G.** **Variability of NRMSE (left) and AIC (right) in the genetic population obtained with *nlme* algorithm by fixing from zero to three parameters as genotype-independent (constant) for 8 growth models (step 2).** The ends of the dashed lines represent the lowest and the highest values still within the 1.5 inter-quartile range of the respectively lower and upper quartile. Circles represent genotypes outliers.

**S1 File. Table F.** **Number of QTLs detected with the parameters of the growth models estimated with *nlme* by fixing from 1 to 3 parameters as genotype-independent**

|  | | |  | **Nb of QTL detected for each parameter** | | | | | | | |  | | |
| --- | --- | --- | --- | --- | --- | --- | --- | --- | --- | --- | --- | --- | --- | --- |
| **Equation** | **Nb of parameters** | **Nb of genotype dependent parameters** | **mu** | | **P3** | **t_0_** | **A** | **B** | **mu_lin_** | **mu_exp_** | **TE** | **Nb of QTL detected** | **Nb of distinct QTL** | **Nb of major QTL** |
| eqGibert | 3 | 3 | 2 | | 2 | _ | 0 | _ | _ | _ | _ | **4** | **2** | **2** |
|  |  | 2 | _ | | 0 | _ | 0 | _ | _ | _ | _ | **0** | **0** | **0** |
|  |  | 1 | _ | | 3 | _ | _ | _ | _ | _ | _ | **3** | **3** | **3** |
| eqKadrani | 4 | 4 | 2 | | 2 | _ | 0 | 0 | _ | _ | _ | **4** | **2** | **2** |
|  |  | 3 | 5 | | 5 | _ | 1 | 2 | _ | _ | _ | **13** | **5** | **3** |
|  |  | 2 | _ | | 1 | _ | _ | 2 | _ | _ | _ | **5** | **3** | **3** |
|  |  | 1 | _ | | 2 | _ | _ | 3 | _ | _ | _ | **5** | **3** | **3** |
| eqLechaudel | 4 | 4 | 1 | | 2 | _ | 0 | 0 | _ | _ | _ | **3** | **2** | **2** |
|  |  | 3 | 2 | | 3 | _ | 3 | 0 | _ | _ | _ | **8** | **4** | **3** |
|  |  | 2 | _ | | 1 | _ | 1 | _ | _ | _ | _ | **2** | **2** | **2** |
|  |  | 1 | _ | | _ | _ | 3 | _ | _ | _ | _ | **3** | **3** | **3** |
| eqHilgert | 4 | 4 | 1 | | 1 | _ | 0 | 0 | _ | _ | _ | **2** | **1** | **1** |
|  |  | 3 | 4 | | 1 | _ | 4 | 0 | _ | _ | _ | **9** | **5** | **3** |
|  |  | 2 | 1 | | 3 | _ | 1 | 2 | _ | _ | _ | **7** | **4** | **2** |
|  |  | 1 | _ | | 3 | _ | 3 | _ | _ | _ | _ | **6** | **3** | **3** |
| eqYin | 3 | 3 | _ | | _ | _ | _ | _ | _ | _ | _ | **_** | **_** | **_** |
|  |  | 2 | _ | | 1 | _ | 3 | _ | _ | _ | 0 | **4** | **2** | **2** |
|  |  | 1 | _ | | 3 | _ | _ | _ | _ | _ | _ | **3** | **3** | **3** |
| EqRichards | 4 | 4 | _ | | _ | _ | _ | _ | _ | _ | _ | **_** | **_** | **_** |
|  |  | 3 | 2 | | 1 | _ | 0 | _ | _ | _ | _ | **3** | **2** | **2** |
|  |  | 2 | _ | | _ | _ | _ | _ | _ | _ | _ | **_** | **_** | **_** |
|  |  | 1 | _ | | _ | _ | _ | _ | _ | _ | _ | **_** | **_** | **_** |
| eqGompertz | 3 | 3 | 0 | | 0 | _ | 0 | _ | _ | _ | _ | **0** | **0** | **0** |
|  |  | 2 | _ | | 0 | _ | 1 | _ | _ | _ | _ | **1** | **1** | **0** |
|  |  | 1 | _ | | _ | _ | _ | _ | _ | _ | _ | **_** | **_** | **_** |
| eqExpolin | 3 | 3 | _ | | _ | 2 | _ | _ | 3 | 2 | _ | **7** | **4** | **2** |
|  |  | 2 | _ | | _ | 0 | _ | _ | 3 | _ | _ | **3** | **3** | **2** |
|  |  | 1 | _ | | _ | 2 | _ | _ | _ | _ | _ | **2** | **2** | **2** |

**S1 File. Figure H.** **Effect of replacing parameters by observed data on the variability of NRMSE (left) and AIC (right) in the genetic population obtained by using the *nlme* algorithm.** The eqLogis.DMmax model is a simplification of eqHilgert model where *DMmax* is explicitly included at the place of *A* and *B* parameters**.** On y-axis NRMSE and AIC are shown. The band inside the box represents the median. The ends of the dashed lines represent the lowest and the highest values still within the 1.5 inter-quartile range of the respectively lower and upper quartile. Circles represent genotypes outliers. One-way ANOVA was performed. Significant growth model effect was observed for NRMSE (*p* = 0.00201) but not for AIC (*p >* 0.05).

**S1 File. Figure I.** **Effect of replacing *P3* parameter by observed data on NRMSE and AIC values. (A) Correlation between NRMSE (upper-left) and AIC (upper-right) values and (B) variability of NRMSE (bottom-left) and AIC (bottom-right) calculated with observed and optimized *P3* by using the *nlme* algorithm for 6 growth models (step 3).** One-way ANOVA was performed. Significant solution effect was observed on AIC (*p* = 0.04), but not on NRMSE (*p* = 0.42).

**S1 File. Table G.** **QTLs detected with parameter solutions of nine growth models obtained by replacing the parameters with average inflection point and final dry mass with *nlme***

| **qtl** | **eq** | **param** | **Nb of param Estimes** | **Nb of param Genot Dependent** | **mk** | **chr** | **pos** | **LOD** | **p-value** | **effect** | **min** | **max** |
| --- | --- | --- | --- | --- | --- | --- | --- | --- | --- | --- | --- | --- |
| qtl_1.1_mu | EqExpolin | mu_lin_ | 2 | 2 | SNP_IGA_2272 | 1 | 0 | 2.95 | 0.0002 | 8.11 | 0 | 7.6 |
| qtl_1.1_mu | EqExpolin | mu_lin_ | 2 | 1 | SNP_IGA_2272 | 1 | 0 | 2.55 | 0.0006 | 7.04 | 0 | 9.7 |
| qtl_1.2_mu | EqExpolin | mu_exp_ | 2 | 1 | FRU | 1 | 31 | 3.6331 | 0.0020 | 4.9475 | 28.1 | 44.7 |
| qtl_1.2_mu | EqHilgert | mu | 3 | 1 | c1.loc34 | 1 | 34 | 2.9525 | 0.0130 | 5.0223 | 0 | 48 |
| qtl_1.2_B | EqHilgert | B | 3 | 2 | c1.loc34 | 1 | 34 | 2.9589 | 0.0160 | 3.7659 | 28.1 | 47.4 |
| qtl_1.2_mu | EqExpolin | mu_lin_ | 2 | 1 | c1.loc34 | 1 | 34 | 3.6707 | 0.0060 | 5.0473 | 28.1 | 44.7 |
| qtl_1.2_mu | EqExpolin | mu_lin_ | 2 | 2 | c1.loc34 | 1 | 34 | 3.4267 | 0.0070 | 6.4113 | 0 | 43.2 |
| qtl_1.2_A | EqKadrani | A | 3 | 2 | c1.loc40 | 1 | 40 | 3.8036 | 0.0030 | 4.3848 | 30.3 | 44.7 |
| qtl_1.2_A | EqKadrani | A | 3 | 1 | c1.loc40 | 1 | 40 | 3.7848 | 0.0010 | 4.2144 | 30.3 | 44.7 |
| qtl_1.2_A | EqLechaudel | A | 3 | 2 | c1.loc40 | 1 | 40 | 3.4670 | 0.0030 | 4.7556 | 30.3 | 44.7 |
| qtl_1.2_A | EqLechaudel | A | 3 | 1 | c1.loc40 | 1 | 40 | 3.3636 | 0.0060 | 4.5649 | 30.3 | 44.7 |
| qtl_1.2_A | EqHilgert | A | 3 | 2 | c1.loc40 | 1 | 40 | 3.5675 | 0.0030 | 4.7162 | 30.3 | 48.9 |
| qtl_4.1_A | EqLechaudel | A | 3 | 3 | SNP_IGA_375010 | 4 | 3.8 | 2.6278 | 0.0490 | 5.1715 | 0 | 28 |
| qtl_4.1_A | EqHilgert | A | 3 | 3 | SNP_IGA_375010 | 4 | 3.8 | 2.6765 | 0.0290 | 5.2068 | 0 | 28 |
| qtl_4.2_mu | EqExpolin | mu_exp_ | 2 | 1 | c4.loc18 | 4 | 18 | 4.5647 | 0.0000 | 3.6961 | 4 | 35.8 |
| qtl_4.2_B | EqHilgert | B | 3 | 2 | PMS40 | 4 | 19.8 | 5.8210 | 0.0000 | 6.2927 | 14 | 27 |
| qtl_4.2_mu | EqHilgert | mu | 3 | 1 | SNP_IGA_387459 | 4 | 19.9 | 5.4102 | 0.0000 | 6.7473 | 14 | 32 |
| qtl_4.2_mu | EqExpolin | mu_lin_ | 2 | 1 | SNP_IGA_387459 | 4 | 19.9 | 5.2278 | 0.0000 | 4.5317 | 14 | 34 |
| qtl_4.2_A | EqKadrani | A | 3 | 2 | SNP_IGA_395013 | 4 | 20.6 | 3.6882 | 0.0030 | 3.6304 | 6.2 | 27 |
| qtl_4.2_A | EqKadrani | A | 3 | 1 | SNP_IGA_395013 | 4 | 20.6 | 3.6423 | 0.0010 | 3.6201 | 6.2 | 29.6 |
| qtl_4.2_A | EqLechaudel | A | 3 | 2 | SNP_IGA_395013 | 4 | 20.6 | 3.0503 | 0.0160 | 3.6592 | 4.3 | 29.6 |
| qtl_4.2_A | EqLechaudel | A | 3 | 1 | SNP_IGA_395013 | 4 | 20.6 | 3.0378 | 0.0100 | 3.6380 | 6.2 | 29.6 |
| qtl_4.2_A | EqHilgert | A | 3 | 2 | SNP_IGA_395013 | 4 | 20.6 | 3.0878 | 0.0070 | 3.7123 | 4.3 | 29.6 |
| qtl_4.2_mu | EqHilgert | mu | 3 | 2 | c4.loc28 | 4 | 28 | 3.1607 | 0.0070 | 8.9188 | 18 | 32.7 |
| qtl_4.2_mu | EqRichardsVfix | mu | 3 | 2 | c4.loc28 | 4 | 28 | 2.5684 | 0.0310 | 6.9211 | 23.4 | 32.7 |
| qtl_4.2_mu | EqExpolin | mu_lin_ | 2 | 2 | CC3 | 4 | 31.4 | 5.7183 | 0.0000 | 5.7527 | 16 | 32.7 |
| qtl_5.1_mu | EqExpolin | mu_exp_ | 2 | 1 | SNP_IGA_549516 | 5 | 8.2 | 2.9703 | 0.0150 | 1.3286 | 2 | 20.2 |
| qtl_5.1_B | EqHilgert | B | 3 | 2 | c5.loc14 | 5 | 14 | 3.0976 | 0.0130 | 1.4673 | 4 | 20 |
| qtl_5.1_mu | EqExpolin | mu_lin_ | 2 | 1 | c5.loc14 | 5 | 14 | 3.0557 | 0.0150 | 1.6341 | 2 | 22 |
| qtl_5.1_mu | EqExpolin | mu_lin_ | 2 | 2 | SNP_IGA_561249 | 5 | 14.6 | 2.8911 | 0.0260 | 2.6432 | 4 | 24 |
| qtl_7.1_B | EqKadrani | B | 3 | 3 | SNP_IGA_761233 | 7 | 14.9 | 2.6501 | 0.0300 | 5.3937 | 4 | 22 |
| qtl_7.1_B | EqLechaudel | B | 3 | 3 | SNP_IGA_761233 | 7 | 14.9 | 2.5812 | 0.0410 | 5.6176 | 2 | 17.4 |
| qtl_7.1_B | EqHilgert | B | 3 | 3 | SNP_IGA_761233 | 7 | 14.9 | 2.5777 | 0.0420 | 5.6285 | 2 | 17.4 |
| qtl_7.2_mu | EqExpolin | mu_lin_ | 2 | 1 | c7.loc32 | 7 | 32 | 2.9633 | 0.0180 | 0.9284 | 24.3 | 41.2 |
| qtl_7.2_mu | EqExpolin | mu_exp_ | 2 | 1 | c7.loc32 | 7 | 32 | 3.2018 | 0.0090 | 2.4302 | 24.3 | 37.9 |
| qtl_7.2_B | EqHilgert | B | 3 | 2 | c7.loc34 | 7 | 34 | 3.0123 | 0.0150 | 0.9900 | 24.3 | 39.5 |

**S1 File. Figure J.** **Representation for 36 genotypes of the values of parameters *mu* and *A* obtained per genotype with *RBGA* and *nlme* algorithms using eqHilgert growth model.** In orange the 20 solutions obtained with *RBGA*. In red the unique solution obtained with *nlme*. In blue the best solution with minimum evaluation value among 20 repetitions with *RBGA*. In green the most different solutions (extreme 1 and extreme 2) obtained from PCA analysis. The background salmon color represents the diversity of all solutions obtained with *RBGA* for the genetic population (161 genotypes x 20 solutions).

**S1 File. Figure K.** **Comparison of NMRSE and AIC for solutions obtained with *nlme* and *RBGA* algorithms for 6 growth models (step 4).** (A) Correlation between NRMSE (left) and AIC (right) for the unique solutions obtained with *nlme* algorithm and the best solutions obtained with *RBGA* algorithm. (B) Variability of NRMSE (left) and AIC (right) for the unique solution from *nlme* algorithm and the three solutions selected from the set of solutions obtained with *RBGA* algorithm. On y-axis NRMSE and AIC are shown. On the x-axis the 4 different solutions are shown. The band inside the box represents the median. The ends of the dashed lines represent the lowest and the highest values still within the 1.5 inter-quartile range of the respectively lower and upper quartile. Circles represent genotypes outliers. One-way ANOVA was performed. Significant solution effect was observed on NRMSE (*p* < 2e^-16^) and AIC (*p* < 2e^-16^).

**S1 File. Figure L.** **Variability of NRMSE (above) and AIC (below) for solutions obtained with *nlme* (uniques) and *RBGA* (Best, Extremes1 and Extremes2) algorithms for 6 growth models (step 4).** On y-axis NRMSE and AIC are shown. On the x-axis the 4 different solutions and 6 equations are shown. The band inside the box represents the median. The ends of the dashed lines represent the lowest and the highest values still within the 1.5 inter-quartile range of the respectively lower and upper quartile. Circles represent genotypes outliers. One-way ANOVA was performed. Significant solution effect was observed on NRMSE (*p* < 0.05) and AIC (*p* < 0.05).

**S1 File. Table H.** **QTLs detected with parameter solutions of ten growth models obtained by parameterizations during the study with *nlme* and *RBGA***
